# The In Vitro Toxicity Profile of ZnS and CdS Quantum Dots in Polysaccharide Carriers (Starch/Chitosan)

**DOI:** 10.3390/ijms25010361

**Published:** 2023-12-27

**Authors:** Anna Grzesiakowska, Magdalena Dzióbek, Marta Kuchta-Gładysz, Joanna Wojciechowska-Puchałka, Karen Khachatryan, Gohar Khachatryan, Magdalena Krystyjan

**Affiliations:** 1 Faculty of Animal Science, University of Agriculture in Krakow, Al. Mickiewicza 21, 31-120 Krakow, Poland; anna.grzesiakowska@urk.edu.pl (A.G.); marta.kuchta-gladysz@urk.edu.pl (M.K.-G.); joanna.wojciechowska-puchalka@urk.edu.pl (J.W.-P.); 2 Faculty of Biotechnology and Horticulture, University of Agriculture in Krakow, Al. Mickiewicza 21, 31-120 Krakow, Poland; magdalena.dzobek@student.urk.edu.pl; 3 Faculty of Food Technology, University of Agriculture in Krakow, Al. Mickiewicza 21, 31-120 Krakow, Poland; karen.khachatryan@urk.edu.pl (K.K.); gohar.khachatryan@urk.edu.pl (G.K.)

**Keywords:** quantum dots, toxicity, nanoparticles, starch, chitosan, cytotoxicity

## Abstract

Nanocomposites are an emerging technology for ensuring food safety and quality. Their unique properties, attributed to nanoparticle presence, facilitate the development of sophisticated sensors and biosensors for detecting harmful substances, microbial growth, and environmental changes in food products. Smart and/or active food packaging development also benefits from the use of nanocomposites. This packaging, or portions of it, provide active protection for its contents and serve as sensors to promptly, simply, and safely identify any detrimental changes in stored food, without elaborate techniques or analyses. Films made from potato starch and chitosan were produced and quantum dots of zinc sulfide (ZnS) and cadmium sulfide (CdS)were synthesized in them for this study. The presence and dimensions of the QDs (quantum dots) were examined with scanning electron microscopy (SEM) and ultraviolet-visible (UV-VIS) spectroscopy. The study aimed to establish the toxicity profile of a starch–chitosan bionanocomposite integrated with ZnS and CdS quantum dots. Cytotoxic and genotoxic features were assessed through cytogenetic instability assessments, consisting of the alkaline comet assay, erythrocyte micronucleus assay, and peripheral blood cell viability analysis of a laboratory mouse model.

## 1. Introduction

The intensive development of nanotechnology observed in recent years has allowed for the development of novel, innovative solutions and materials. The unique properties of nanomaterials, resulting from their reduced size, allow them to be widely used in various industries, including biomedicine, pharmaceuticals, electronics, and optics [1,2,3]. New developments have made it possible to obtain quantum dots, or smaller semiconductor materials, crystals of 2–10 nm in size, with unique chemical and physical properties and photoluminescence capabilities. The color of the emitted light depends on the size of the crystal [3,4,5]. Quantum dots consist of elements belonging to groups III–V, II–VI, IV–VI of the periodic table [2,6]. The most widespread applications are cadmium-based quantum dots, such as CdS, CdSe, CdS/ZnS, CdSe/ZnS, but also carbon dots [2]. However, under less favorable conditions, the core structure can degrade, increasing the release of metals in ionic form, which can result in greater toxicity of these materials. To reduce ion leakage from the QD structure, biocompatible and non-toxic materials are used in the form of coatings, such as ZnS [3,7,8,9]. ZnS is commonly used to increase QD fluorescence efficiency and stabilization, as well as to reduce the toxicity of the reactive core [7].

In order to increase the bioavailability and use of quantum dots in other industries as well, more environmentally friendly carriers are being developed. Such carriers are polysaccharide nanocomposites, which are hydrophilic and provide a good barrier against oxygen and carbon dioxide. However, poor extensibility and barrier properties against water vapor prevent their full utility as food packaging. Improving the properties of polysaccharide composites is possible by introducing a nanoscale additive, including precisely quantum dots with unique chemical properties [10,11,12]. The most commonly used for the preparation of biodegradable food packaging are starch, chitosan, alginate, and cellulose, due to their easy availability, low production cost, and unique properties [1,10,11,12].

Starch is a natural, renewable, biocompatible, and biodegradable plant polymer. It has found wide application with food processing, paper, and textile industries. However, starch itself has limited solubility, poor functional properties, and poor tolerance to processing under various conditions, which limits its use as a potential food packaging [10,13,14]. The development of starch nanoparticles (SNPs) allows for a reduction in particle size while maintaining a relatively large active surface area, acquiring unique properties, used in biomedicine, among other applications [14,15]. The second polysaccharide commonly used as a biological carrier for nanomaterials is chitosan. It is a natural cationic and hydrophilic polymer obtained by the alkaline hydrolysis of chitin, and is also non-toxic and biocompatible. Chitin itself is an amino polysaccharide naturally extracted from fungal cell walls [16]. Nanoparticles based on chitosan are characterized by good antimicrobial properties [17], and thus, they have found wide application in biomedical components, including drug carriers [18], biosensor materials [19], and tissue engineering/regeneration [1,2,20,21,22,23]. Due to the ability of chitosan nanoparticles to penetrate biological barriers directly into cells, this can induce its toxic effects on human cells [2]. It has been shown that chitosan nanoparticles can reduce cell viability, disrupt cell proliferation, or compromise cell membrane integrity [24]. The potential toxicity of chitosan and chitosan nanoparticles depends on the degree of acetylation and molecular weight [16,25,26].

Both polysaccharides present a number of beneficial properties, allowing them to be widely used. The creation of a polymer based on starch and chitosan has made it possible to obtain a biological, biodegradable food packaging with antibacterial and antifungal properties. However, in order to improve its storage properties, it is necessary to enrich it with nanoadditives, such as graphene oxide [12], or CdS and ZnS quantum dots [11]. When producing films and biopolymers through green synthesis methods, it is important to keep in mind the fact of the potential release of their components and them getting into food (e.g., Cd^2+^, Zn^2+^), and thus into the human body. There is a lack of thorough research related to the effects of these agents on the human body, despite numerous studies defining the nature of each of the compounds: starch, chitosan, ZnS QD, CdS QD. Rather, with the exception of the last, these compounds are considered non-toxic [1,2,14]. Due to the small size of the particles and their use in the biomedical industry as drug carriers and biosensors, and thus their ability to cross cellular barriers and bioaccumulate in organs, concern over potential human exposure seems important, including when these materials are used as food packaging. Therefore, in order to expand the information on the toxic profile of the compounds, it seems necessary to conduct further studies, including studies on material from laboratory animals, characterized by similarities in structure and organ function [27]. The purpose of the study was to develop the toxicity profile of a starch-chitosan biocomposite with the addition of cadmium sulfide and zinc sulfide quantum dots as potential food packaging. Cytotoxic and genotoxic properties were evaluated using the following cytogenetic instability assays: alkaline comet assay and erythrocyte micronucleus assay, as well as peripheral blood cell viability analysis of a laboratory mouse as a model organism.

## 2. Results and Discussion

To develop the toxicity profile of starch–chitosan films with ZnS and CdS quantum dots, a cell viability assessment and two cytogenetic tests were performed: an alkaline variant comet assay and erythrocyte micronucleus assay, in in vitro conditions.

### 2.1. Cell Viability Assessment Test

**Figure 1:**
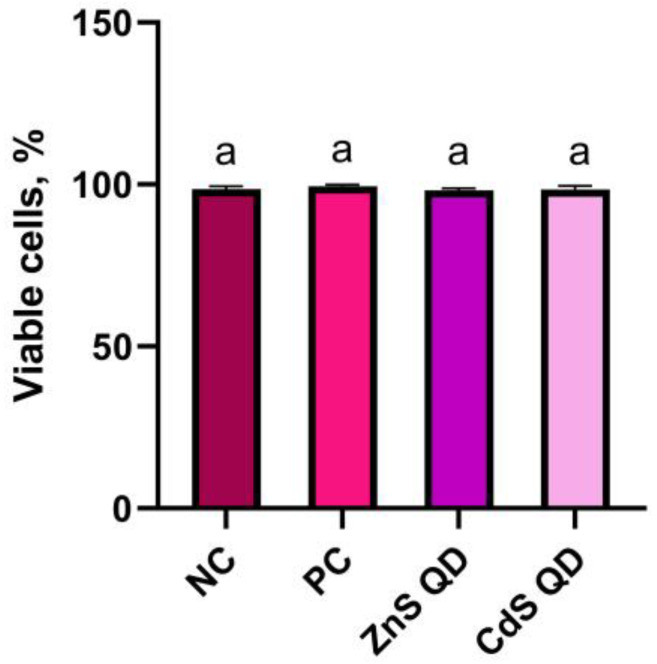
Viability of mouse peripheral blood cells after exposure to test films: NC—negative control; PC—positive control; ZnS QD—ZnS QD film; CdS QD—CdS QD film. The values on the graph represent the mean and standard error; a—averages between groups marked with different letters are significantly different (*p* ≤ 0.05).

In the experiment, the viability of mouse peripheral blood cells was evaluated in different experimental groups: a negative control, a pure blood sample stored for 1 h, cells exposed for 1 h to a control film, and cells exposed for 1 h to bionanocomposites with QD ZnS or CdS. Evaluation of cell viability was carried out in a Burker chamber, after treating cells with trypan blue. The dye penetrates into dead cells, due to a change in the integrity of the cell membrane, and the cells themselves are characterized by a blue staining, while viable cells remain colorless [28]. Based on the results obtained, there was no damaging effect of the tested materials on the viability of mouse peripheral blood cells. Detailed data are shown in Figure 1. The enrichment of starch–chitosan films with zinc sulfide or cadmium sulfide quantum dots did not affect the viability of the tested cells in any way. The results obtained from the analysis of the animal material allow us to conclude the suitability of the bionanocomposite as a potential material used for the protection and storage of food products.

The viability of mouse peripheral blood cells at similar levels was observed by Krystyjan et al. [12] after 24 h exposure to a starch–chitin biocomposite and when this composite was enriched with the addition of graphene oxide. Banu et al. [29] in their in vivo study of the effects of ZnSO_4_ on mouse leukocytes found no effect of the compound on cell viability. Cell viability in all groups was in the 94–96% range. Manzoor et al. [30] found no effect of ZnS QD on the viability of mouse fibroblast cells (L929). They considered ZnS QD to be a non-toxic material for humans. Even the potential release of Zn in ionic form from ZnS, according to the authors, would not adversely affect mammalian cells, due to the biological role of Zn in cells and the body. Li et al. [9] compared the effects of ZnS QD and CdS QD on human endothelial cells in their study. Viability analysis, assessed with trypan blue, showed no effect of ZnS QD on cell proliferation. The absence of toxicity of these quantum dots on the endothelial cells tested was found not to depend on the concentration tested and the particle coating. CdS QDs at a higher concentration, 10 µM, showed pronounced toxic effects on human endothelial cells, inducing significant cell death. In our study, there were no differences in the viability of cells treated with the two types of quantum dots. The determining factor was the carrier of the dots, i.e., the starch–chitosan biocomposite, and the concentration of ZnS and CdS.

### 2.2. Comet Assay

**Figure 2:**
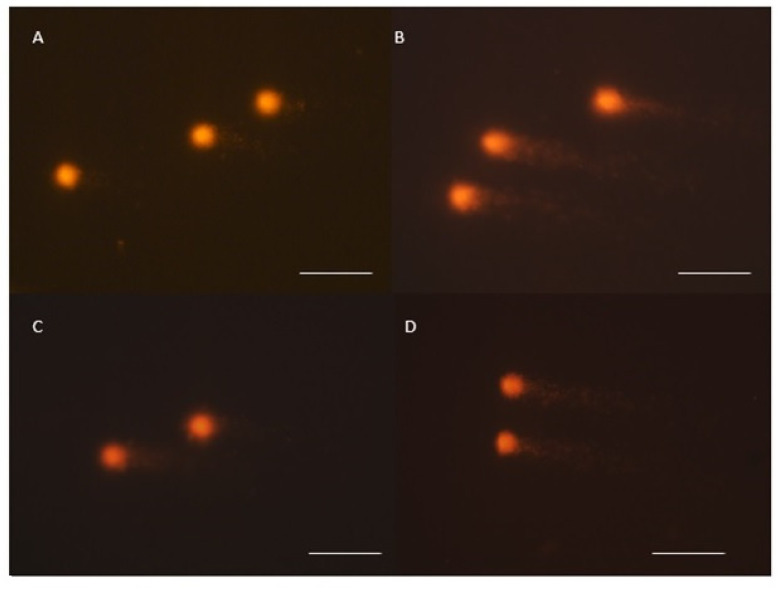
Peripheral blood cells analyzed in comet assay after exposure to biocomponents: (**A**)—cell from negative control; (**B**)—cell from positive control; (**C**)—cell exposed on CdS QD film; (**D**)—cells exposed on ZnS QD film. Magnification 400×. Scale bar 10 µm.

**Figure 3:**
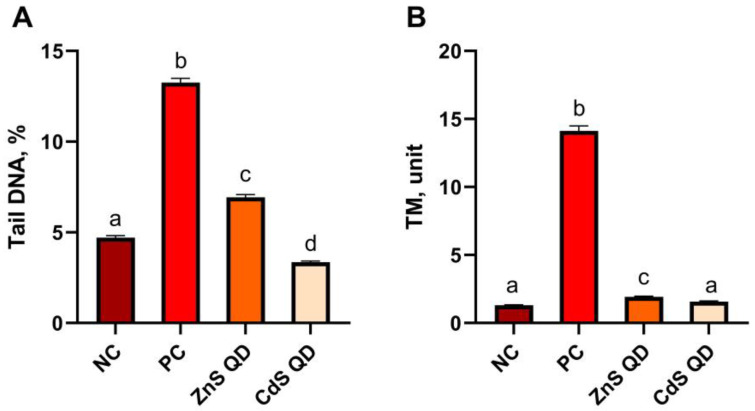
Toxicity of the analyzed composites evaluated by comet assay based on the values of the parameters tail DNA % (**A**) and TM (**B**): NC—negative control; PC—positive control; ZnS QD—ZnS QD film; CdS QD—CdS QD film; The values on the graph represent the mean and standard error; a, b, c, d—averages between groups marked with different letters are significantly different (*p* ≤ 0.05).

The toxicity of the tested films was evaluated by a comet assay performed in the alkaline variant (Figure 2 and Figure 3). It is one of the basic methods of assessing DNA fragmentation, and is used to determine the degree of sensitivity of cells, an individual to a specific genotoxic agent, but also allows one to assess the level of DNA repair and the efficiency of repair mechanisms. The comet assay allows for the identification of single-stranded and double-stranded DNA breaks and other cellular modifications that could develop into a break. Analysis of cellular damage is possible after electrophoretic separation of DNA and appropriate staining of cells. In a microscopic image, cells with disrupted DNA integrity by their shape are similar to a comet, the head of which is the cell nucleus, and the tail—damaged fragments of DNA strands [31,32,33]. For this purpose, damage to 1000 cells in each experimental group and a total of 4000 mouse peripheral blood cells was analyzed. The main parameter indicating the toxicity of the tested biocomposites on mouse somatic cells was the percentage of DNA in the comet tail (% tail DNA). The average value of this parameter in the negative control was 4.71 ± 0.12% of DNA in the comet tail, and by comparison, in the positive control, cell damage increased to a level of 13.25 ± 0.24% DNA after treatment with the starch–chitosan composite alone. The values of the % tail DNA parameter obtained for mouse peripheral blood cells between the control groups differed significantly (*p* ≤ 0.05); the results are shown in Figure 3A. The addition of ZnS quantum dots in the bionanocomposites induced a significant increase in somatic cell damage to an average level of 6.93 ± 0.15% tail DNA. In contrast, CdS QDs showed a different, more protective effect on mouse peripheral blood cells, as the level of DNA degradation in this group was the lowest, even compared to the negative control, at only 3.35 ± 0.07% tail DNA. The value of the % tail DNA comet parameter differed significantly between all the groups analyzed.

The second parameter evaluated in the comet test, indicating the toxicity of the tested materials, was the tail moment (TM). This is a complementary parameter without a unit, and is derived as the product of the percentage of DNA in the comet’s tail and the length of the tail. The value of this parameter for the negative control was 1.30 ± 0.04 TM, and for the experimental groups with the addition of quantum dots, 1.92 ± 0.05 for ZnS QD and 1.58 ± 0.05 for CdS QD, respectively. A significantly higher average tail moment was characterized by somatic cells after treatment with the biocomposite as a positive control, TM at 14.12 ± 0.38. Significant differences were found between the obtained TM values for the analyzed groups at *p* ≤ 0.05, except for the comparison of TM for the negative control and CdS QD (Figure 3B).

Analysis of the toxicity profile of the starch–chitosan biocomposite with graphene oxide by Krystyjan et al. [12] showed a higher degree of damage by the pure control composite in the comet test. Damage to somatic cells in this case was estimated at 16.26 ± 12.14% of DNA in the comet tail after 24 h exposure. In the current study, damage induced by the control composite was at a slightly lower level after short-term exposure. Liu et al. [34] conducted genotoxicity studies of cadmium sulfide quantum dots on human peripheral blood lymphocytes. In the experiment, they used two types of quantum dots: uncoated CdS QDs and CdS QDs surrounded by thioglycolic acid. They evaluated toxicity using a comet assay and a micronucleus assay. In the comet assay, they evaluated the effect of 6 h exposure of peripheral blood cells. They showed that both forms of CdS QD tested induced DNA damage, as the percentages of DNA content in the comet tail were higher than the average for the control group (20%). They estimated DNA damage in human lymphocytes at >30% tail DNA after CdS QD treatment and as being 25% after CdS QD treatment with thioglycolic acid. At the same time, based on the second parameter, tail length, they indicated greater toxicity of pure CdS QDs. As a mechanism for this toxicity, they pointed to the ability of QDs to infect cells by endocytosis, and their subsequent effects in cells by generating ROS and oxidative stress, lipid peroxidation, and subsequent DNA damage [34]. Many studies on metal quantum dots use an additional core coating of ZnS layer to increase photostability and reduce metal efflux from the core, which would reduce the potential toxicity of QDs [2,6]. The inhalation toxicity of Zn salts, specifically ZnSO4, on mouse leukocytes was studied by Banu et al. [29] using an alkaline variant of the comet assay. They found that the level of DNA damage induced by ZnSO_4_ was proportional to the dose of the compound and inversely proportional to the exposure time. Sharif et al. [35] showed in a comet assay that zinc at doses of 4 and 16 µM reduced DNA strand breaks of human lymphoblastoid cells, while at higher concentrations, they observed an increase in single-strand DNA damage, indicating the potentially genotoxic nature of zinc sulfide.

### 2.3. Erytrocyte Micronucleus Assay

**Figure 4:**
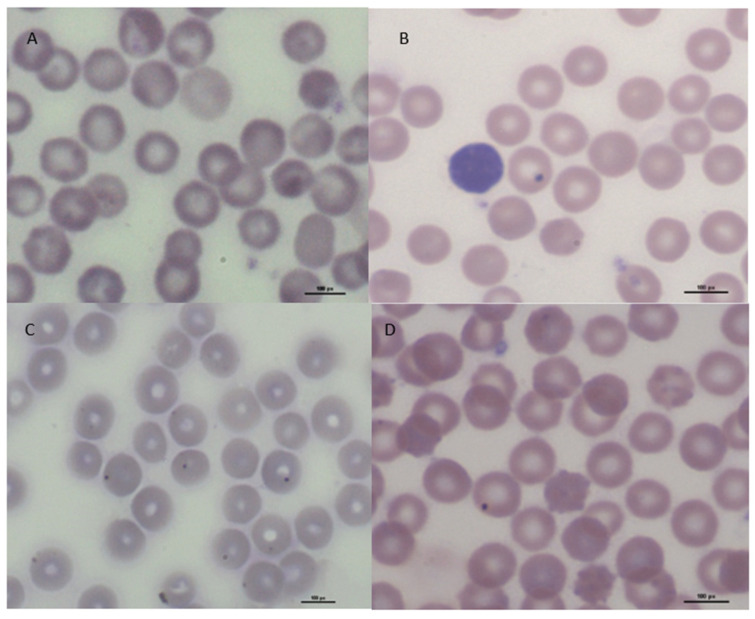
Peripheral blood cells analyzed in erythrocyte micronucleus assay after exposure to biocomponents: (**A**)—cell from negative control; (**B**)—cell from positive control; (**C**)—cell exposed on CdS QD film; (**D**)—cells exposed on ZnS QD film. Magnification 1000×. Scale bar 100 µm.

Another method to assess the toxicity of nanomaterials in vitro is the erythrocyte micronucleus assay. This method analyzes the presence of micronuclei in immature erythrocytes (PCE, polychromatic erythrocytes) from peripheral blood, which are in the final stage of erythropoiesis. Proliferating cells were treated with the tested nanocomposites, and the resulting potential damage in the form of damaged chromosomes or chromatids was observed in the cytoplasm of the cells as micronuclei (Howell–Jolly bodies). Identification of micronuclei in PCE is possible due to their different staining compared to mature, normochromatic erythrocytes (NCE). NCE erythrocytes stain pinkish-yellow, while PCEs, due to their lower hemoglobin content and RNA still present in the cell, show a pinkish-purple, pinkish-blue pigmentation [36,37]. Examples of analyzed cells in each experimental group are shown in microphotographs Figure 4A–D.

**Figure 5:**
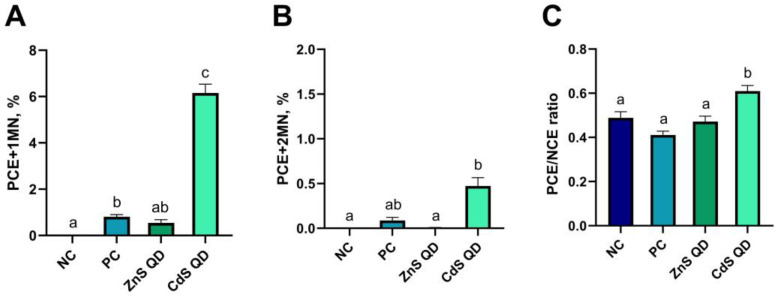
Toxicity of the analyzed composites evaluated by erythrocyte micronucleus assay based on the values of the parameters PCE + 1 MN % (**A**), PCE + 2 MN % (**B**) and PCE/NCE (**C**): NC—negative control; PC—positive control; ZnS QD—ZnS QD film; CdS QD—CdS QD film. The values on the graph represent the mean and standard error; a, b, c—averages between groups marked with different letters are significantly different (*p* ≤ 0.05).

The presence of micronuclei (1 or 2, and their proportion was determined as a percentage) was analyzed in the cells, and in addition, the PCE/NCE ratio, which is considered an indicator of the cytotoxicity of the test agent, was calculated for each experimental group. In peripheral blood cells derived from the negative control, no micronuclei were found in PCE, and the ratio of immature to mature erythrocytes was determined to be 0.49 ± 0.03. Peripheral blood erythrocytes treated with starch–chitosan film, a positive control, were characterized by the presence of 0.81 ± 0.09% of PCE + 1MN and 0.09 ± 0.03% of PCE + 2 MN, and the PCE/NCE value for this group was 0.41 ± 0.02. Between the control groups, there were significant differences only in the percentage of PCE with one micronucleus (Figure 5A–C). Peripheral blood cells exposed to the biocomposite with ZnS quantum dots showed the presence of 0.54 ± 0.14% PCE + 1MN, while no two micronuclei were observed in cells from this group. The presence of cadmium sulfide quantum dots in the biofilm induced chromatin damage, observed as an increase in the number of micronuclei in immature erythrocytes. The percentage of PCE with 1 micronucleus in this experimental group was 6.15 ± 0.38, and two micronuclei at 0.47 ± 0.09% in PCE. The values of both of these ratios for the CdS QD group differed significantly compared to all other groups. The PCE/NCE ratio was determined at 0.61 ± 0.03 in this group and also showed significant differences with respect to the control and ZnS QD groups.

Higher values of erythrocyte micronucleus assay parameters were demonstrated only for the group of somatic cells treated with CdS QD biocomposite. According to Aye et al. [38], cadmium ions, through three different mechanisms, induce DNA strand breaks in mammalian cells. They pointed to the generation of oxygen free radicals, and thus oxidative stress by cadmium as the main mechanism. This team’s study showed that nanoparticle cadmium and cadmium salts reacted with cellular structures, but their mechanism of action was not the same. QDs showed weaker effects compared to CdCl_2_, while they showed higher photoinduced genotoxicity. Using a micronucleus assay, Grzesiakowska et al. [33] demonstrated the toxic effects of QDsN, observed as a significant increase in the occurrence of micronuclei in cells. Liu et al. [34] conducted a 72 h exposure of human lymphocytes to CdS QDs in a micronucleus assay. The frequency of identified micronuclei was higher for both CdS QD and its thioglycolic acid-coated version. However, a significantly higher presence of micronuclei in lymphocytes was demonstrated after treatment with CdS QDs alone (5‰). As a mechanism of toxicity related to chromosome damage, the authors pointed to the potential ability of quantum dots to inhibit, disrupt, or interrupt cell division. They also showed that surface modification of QDs can effectively retard their harmful effects, but their exact mechanisms of genotoxicity is unknown [34]. Sharif et al. [35] based on the extremely important biological role of zinc in cells, as a factor affecting cell proliferation, apoptosis, and the development of the immune system, conducted an evaluation of the effects of Zn supplementation or the absence of Zn on human lymphoid cells. Analysis by the CBMN variant micronucleus assay showed that zinc-deficient cells exhibited a higher frequency of micronuclei, nucleoplasmic bridges, and nuclear buds in the cells, while a reduction in DNA damage was observed compared to supplemented cells [35]. Zinc-based quantum dots are considered environmentally friendly with negligible toxicity to living organisms, especially at low concentrations [4,5]. Manzoor et al. [30] evaluated the cytotoxicity of ZnS QD and CdS QD using the MTT assay. They showed that even a high dose (100 µM) of ZnS QD and 48 h exposure did not cause toxic effects on various cell lines, including normal mouse lung fibroblast cells and carcinogenic lines. In contrast, under the same conditions, CdS QDs showed high levels of toxicity [30].

## 3. Materials and Methods

### 3.1. Materials

The nanocomposites were produced through the use of certain chemical reagents, including potato starch (with an amylose to amylopectin ratio of 26:74, 12% moisture, Sigma-Aldrich, Poznan, Poland), high-molecular-weight chitosan (310,000–375,000 Da, degree of deacetylation >75%, from shrimp shells, Sigma-Aldrich, Poznań, Poland), acetic acid (99.5–99.9%, pure for analysis, Pol-Aura, Morąg, Poland), glycerol (pure for analysis, EuroChem BGD, Tarnów, Poland), zinc acetate (99.999% trace metals basis, Sigma-Aldrich, Poznań, Poland), ammonium sulfide (20 wt. % in H_2_O Sigma-Aldrich, Poznań, Poland), and cadmium acetate (≥99.99% trace metals basis, Sigma-Aldrich, Poznań, Poland).

### 3.2. Preparation of Nanocomposite Films

**Figure 6:**
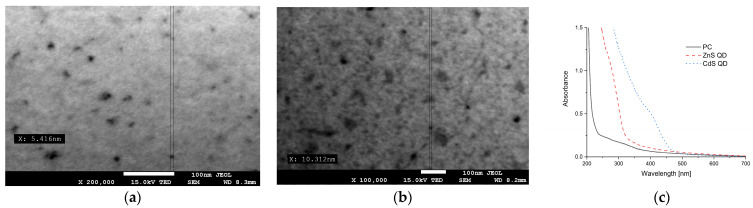
Scanning electron microscopy images of ZnS QD (**a**), CdS QD (**b**), and UV-Vis spectra of control film (PC) and nanocomposite films (**c**).

The nanocomposite films were produced following the method described by Grzebieniarz et al. [11]. Given that biopolymers can exhibit slight property variations depending on the batch, UV-Vis spectra were conducted for all films, and electron microscopy was employed for the nanocomposite ones to ensure repeatability and confirm the results. Electron microscopy images (Figure 6) demonstrate the successful synthesis of ZnS (Figure 6a) and CdS (Figure 6b) nanoparticles with average diameters of 5 and 10 nm, respectively. Additionally, UV-Vis spectra (Figure 6c) confirm the presence of ZnS and CdS nanoparticles and are consistent with previous results [11]. The concentrations of quantum dots in the resulting composites are 0.518% and 0.767% for ZnS QD and CdS QD, respectively.

### 3.3. Scanning Electron Microscopy

The sizes and shapes of the prepared nanoparticles were analyzed using the high-resolution JEOL 7550 scanning electron microscope, which was equipped with a TEM detector (Akishima, Tokyo, Japan).

### 3.4. UV-VIS Spectroscopy

The UV-Visible absorption spectra of the nanocomposite films were analyzed using a Hitachi U2900 spectrophotometer (Hitachi Co. Ltd., Tokyo, Japan) with quartz cuvettes in the 200–700 nm range.

### 3.5. Toxicity Profile

Toxicity assessment of the composites was performed on freshly collected peripheral blood from 10 wild-type Wistar (WT) mice. Under Poland’s current regulations on animal research, experiments conducted on the blood of slaughtered animals do not require the approval of the Local Ethics Committee. Viability analysis and a comet assay were used to assess toxicity. For this purpose, whole peripheral blood cells were exposed to control (starch- and chitosan-based bionanocomposites) and bionanocomposites with QD-ZnS and QD-CdS. Two sterilized discs cut from the tested composites were placed at the bottom of a sterile Eppendorf tube mixer and 150 µL of whole peripheral blood and 50 µL of RPMI-1640 culture medium (Sigma Aldrich, Poznan, Poland) were pipetted. Short-time exposure was carried out for 1 h at room temperature. The negative control consisted of blood samples not exposed to the tested composites—clean blood, used at 0 h immediately after collection stored with RPMI 1640 medium (Roswell Park Memorial Institute 1640 Medium, Roswell Park Comprehensive Cancer Center, Buffalo, NY, USA) for 1 h at room temperature.

### 3.6. Viability Assessment

Cell viability was assessed by staining with 0.4% trypan blue solution. Ten microliters of whole peripheral blood and ten microliters of 0.4% trypan blue (Sigma-Aldrich, Poznan, Poland) were mixed on a microscopic slide and incubated for 2 min at room temperature. The 10 µL were then transferred to a Bürker chamber. Live cells—unstained and dead cells stained blue—were counted in three large squares under the Bürker chamber.

### 3.7. Alkaline Comet Assay

The evaluation of changes in nuclear DNA integrity in somatic cells was performed according to the comet assay protocol of Singh et al. [39] with modification. The 10 µL whole peripheral blood suspended in 75 µL of LMP agarose (low melting point) (Sigma-Aldrich, Poznan, Poland) was applied to microscopic slides coated with 75 µL NMP agarose (normal melting point) (Sigma-Aldrich, Poznan, Poland). Lysis of the slides was carried out for 1 h in alkaline buffer (2.5 M NaCl (Sigma-Aldrich, Poznan, Poland), 0.1 M EDTANa2 (ethylenediaminetetraacetic acid disodium salt dihydrate) (Sigma-Aldrich, Poznan, Poland), 10 mM TRIS (Trizma base) (Sigma-Aldrich, Poznan, Poland) and 1% Triton X-100, pH = 10 (Sigma-Aldrich, Poznan, Poland)) at +4 °C with limited light. Electrophoresis was conducted under alkaline conditions in 30 mM NaOH buffer (Sigma-Aldrich, Poznan, Poland) with 2 mM EDTANa2, pH = 12.5 (Sigma-Aldrich, Poznan, Poland), under limited light for 20 min at 0.6 V/cm. Neutralization was carried out in 0.4 M Tris (Sigma-Aldrich, Poznan, Poland). For detection, slides were stained with ethidium bromide at a concentration of 200 µg/mL. Microscopic documentation was performed using a Zeiss Imager A2 epifluorescence microscope with AxioCam MRc5 software (NIS-Elements image analysis software ver. F2.31, Carl Zeiss, Jena, Germany). Lymphocyte damage assessment was performed using CASP 1.2.3b software (ZapsLab, CaspLab.com, Wroclaw, Poland). For each animal, 100 comets were analysed in each of four experimental groups. The parameter determining the toxicity profile in the comet assay was the percentage of DNA in the tail (% of DNA in the tail, TD %) and the tail moment.

### 3.8. Erytrocyte Micronucleus Assay

The 5 μL of whole peripheral blood and 5 μL of phosphate-buffered saline (PBS, Sigma Aldrich) were spotted onto microscopic slides, and smears were made, two replicates per individual, in each of four experimental groups. Slides were fixed with methanol (CZDA, Avantor Performance Materials Poland S.A., Gliwice, Poland) for 10 min. The smears were stained using the May–Grünwald–Giemsa method. The obtained slides were subjected to microscopic analysis using a Jenaval Carl Zeiss light microscope (Carl Zeiss, Jena, Germany). Analysis was performed at 1000× magnification using immersion oil. For each specimen, from each individual in each of four experimental groups, 2000 immature erythrocytes—PCEs (1000 PCEs per replicate, 2 slides per specimen/individual)—were counted, including those containing one and two micronuclei, as well as mature erythrocytes—NCEs. Photographic documentation was made using a Nikon camera (Nikon, Tokyo, Japan) and Imaging Software NIS-Elements F2.1 (Nikon, Tokyo, Japan).

### 3.9. Statistical Analysis

All results are expressed as means with standard error. Data were checked for normality using the Shapiro–Wilk test and for homogeneity of variance using Levene’s test. For data that did not have a normal distribution, a log transformation was applied to attain normality. For normally distributed data, a one-way analysis of variance (ANOVA) with Tukey’s post hoc test was used. For data that did not have a normal distribution and equality of variance, a nonparametric Kruskal–Wallis test with Dunn’s multiple comparisons test was used. For all tests, a probability of *p* ≤ 0.05 was considered statistically significant. Analyses were conducted using Statistica 13.0 software.

## 4. Conclusions

The low degree of toxicity of the analyzed materials on mouse somatic cells allows us to conclude the suitability of the tested bionanocomposite as a potential material for the preservation and storage of food products. The properties allowing one to extend the storage life and usefulness of biological products (including potentially food products) were confirmed by the high survival rate of the tested cells for all examined composites. The starch–chitosan composite itself showed greater disruption of the nuclear chromatin integrity of mouse somatic cells, especially in the comet assay. In the erythrocyte micronucleus test, the film with cadmium sulfide quantum dots had a higher destructive effect, in the form of induction of micronuclei. Nuclear chromatin damage observed in mouse somatic cells as a result of exposure to the tested nanocomposites was less than 7% loss of DNA from the cell nucleus and up to 6% PCE with one micronucleus. In order to improve the properties of the starch–chitosan composite, it is recommended that it should be enriched with zinc sulfide quantum dots, which were characterized by lower levels of induced nuclear chromatin damage in laboratory mouse somatic cells in the study.

## Data Availability

The data presented in this study are available on request from the corresponding author.
